# Nutritional intervention in patients with hypopituitarism secondary to pituitary adenomas with metabolic syndrome

**DOI:** 10.1186/1758-5996-7-S1-A118

**Published:** 2015-11-11

**Authors:** Renata Elisabeth Rothen, Bruna Breyer de Freitas, Carolina Garcia Soares Leães, Miriam da Costa Oliveira, Fernanda Michielin Busnello, Júlia Fernanda Semmelmann Pereira-Lima

**Affiliations:** 1 UFCSPA, Porto Alegre, Brazil

## Background

The deficiency in the production of any of the pituitary hormones, denominated hypopituitarism, may cause the increase of visceral fat, dyslipidemia and decrease of muscle mass. Metabolic syndrome (MetS) is a complex disorder, characterized by alterations of the lipid profile, abdominal obesity, blood pressure and insulin resistance, being highly prevalent in hypopituitarism.

## Objective

We performed clinical, laboratory and nutritional assessment in patients with hypopituitarism, pituitary adenomas and MetS, before and six months post nutritional intervention and compared outcomes.

## Materials and methods

this was a cross-sectional study of 36 outpatients, aged 20-75 yrs., with MetS whose diagnosis was established based on the International Diabetes Federation criteria and hypopituitarism, in the presence or after pituitary adenoma treatment. In the anthropometric assessment the body weight, height, body mass index (BMI) and waist circumference (WC) were measured. Furthermore, serum lipid levels and fasting glucose were measured. Nutritional assessment included sociodemographic information, historic of diseases, medications as well as food intake by a 24-h food recall. These were evaluation prior and post nutritional intervention, on initial visit (V0) and final visit (V6) after six months.

## Results

Nineteen women and 17 men were studied, aged 29-73 yrs. (56.9±9.6), 21 of them presented clinically non-functioning pituitary adenoma, 9 prolactinoma, 4 somatotropinoma, and 2 adrenocorticotropinoma. With respect to hypopituitarism, 28 patients presented panhypopituitarism and 8 isolated hormone deficiency. Mean body mass index on V0 was of 32,9±5,89 kg/m^2^ and 50% with class I obesity, on V6 was of 31,8±5,8 kg/m^2^ and 44,4% with class I obesity. After nutritional intervention, patients presented a decrease in weight, BMI and WC (p=<0.001). Food intake was characterized by low intake of energy and fiber, adequate intake levels of carbohydrate and fat, and high intake of proteins on pre and post intervention. After intervention, there was a decrease on total cholesterol (p=0.014) and LDL cholesterol (p=0.030), with cholesterol levels, coming to normal.

## Conclusions

Nutritional intervention in patients with hypopituitarism, pituitary adenomas and MetS proved to be effective, leading to decreased anthropometric parameters of weight, IBM, WC, as well as biochemical parameters of total and LDL cholesterol.

